# How long is the last mile? Evaluating successful malaria elimination trajectories

**DOI:** 10.1186/s12936-022-04368-3

**Published:** 2022-11-14

**Authors:** Justin M. Cohen, Deepika Kandula, David L. Smith, Arnaud Le Menach

**Affiliations:** 1 Clinton Health Access Initiative, Boston, USA; 2 Institute for Health Metrics and Evaluation, University of Washington, Seattle, USA

**Keywords:** Malaria, Elimination, Malaria incidence, History, Goal setting

## Abstract

**Background:**

Many national malaria programmes have set goals of eliminating malaria, but realistic timelines for achieving this goal remain unclear. In this investigation, historical data are collated on countries that successfully eliminated malaria to assess how long elimination has taken in the past, and thus to inform feasible timelines for achieving it in the future.

**Methods:**

Annual malaria case series were sought for 56 successful elimination programmes through a non-systematic review. Up to 40 years of annual case counts were compiled leading up to the first year in which zero locally acquired or indigenous cases were reported. To separate the period over which effective elimination efforts occurred from prior background trends, annual case totals were log transformed, and their slopes evaluated for a breakpoint in linear trend using the *segmented* package in R. The number of years from the breakpoint to the first year with zero cases and the decline rate over that period were then calculated. Wilcox-Mann-Whitney tests were used to evaluate whether a set of territory characteristics were associated with the timelines and decline rates.

**Results:**

Case series declining to the first year with zero cases were compiled for 45/56 of the candidate elimination programmes, and statistically significant breakpoints were identified for 42. The median timeline from the breakpoint to the first year with zero local cases was 12 years, over which cases declined at a median rate of 54% per year. Prior to the breakpoint, the median trend was slightly decreasing with median annual decline of < 3%. Timelines to elimination were fastest among territories that lacked land boundaries, had centroids in the Tropics, received low numbers of imported cases, and had elimination certified by the World Health Organization.

**Conclusion:**

The historical case series assembled here may help countries with aspirations of malaria elimination to set feasible milestones towards this goal. Setting goals for malaria elimination on short timescales may be most appropriate in isolated, low importation settings, such as islands, while other regions aiming to eliminate malaria must consider how to sustainably fund and maintain vital case management and vector control services until zero cases are reached.

## Background

Many national malaria programmes have recently set time-bound goals to eliminate malaria [1], but it is currently unclear what timelines such accomplishments will truly require. Of 21 countries identified in 2016 as potential contenders for elimination by 2020 [2], only seven succeeded in interrupting transmission by the deadline, while the elimination goal for all others was shifted back another five years [3]. One investigation found that targets set by sub-Saharan African malaria programmes generally were more aligned with global goals than local contexts, and of 135 targets set, only four were achieved [4]. Setting ambitious goals is laudable, but there is a risk that setting and then missing aspirational, infeasible goals will lead to cynicism and mistrust from the afflicted communities and potentially jeopardize future funding from fatigued donors.

The final years of an elimination campaign may present the greatest political and operational challenges of the entire endeavour [5, 6], so an evidence-based understanding of how long that effort will be is critical for realistic planning, budgeting, and expectation setting. During the Global Malaria Eradication Program of the 1950-1960 s, elimination was codified as involving total coverage with indoor residual spraying for “about four years” [7] – though potentially upwards of seven [8] – followed by a “consolidation” phase of intensive surveillance and response until no indigenous cases had been observed for three years [8]. These timelines were supported by mathematical modelling suggesting that total coverage with effective attack measures could reduce the reproductive rate sufficiently for malaria prevalence to fall to < 20% of its original value within one year, and to < 3% within two [9]. Those models subsequently have been extended to include more complex dynamics, such as superinfection, which increase expected timelines to reach a prevalence of 1% by 2–3 years beyond those estimated by the Ross-Macdonald model, and timelines from 1% to zero may be longer still [10].

Today, despite extensive guidance on how elimination should be pursued [11–13], empirical data are lacking on how long a successful elimination programme will take. In this investigation, historical data are collated on countries that successfully eliminated malaria to assess how long elimination has taken in the past, and thus to inform feasible timelines for achieving it in the future. By examining the time to elimination and the rate at which incidence typically has declined over time, factors that may speed or slow it are also assessed.

## Methods

### Identifying countries that eliminated malaria

Countries that observe zero indigenous malaria cases for three consecutive years can request World Health Organization (WHO) certification of malaria elimination, which is awarded based on evidence that indigenous cases are no longer occurring, surveillance is strong enough to identify them if they were, and the country has sufficient means to sustain the achievement [14]. The WHO maintains a registry of countries that have been certified [15]. To date, 38 countries or regions have received this designation: 14 in the 1960 s (counting the republics of the former federation of Yugoslavia as a single certification event), 11 in the 1970-80 s, none in the 1990 s, and 13 since 2000.

In addition to the 38 certified countries, the WHO maintains a supplementary list intended to catalog countries or regions that were always free of malaria or where it “disappeared without specific measures”. This list currently includes 61 countries or regions [15]. Criteria for inclusion on the list are not clear as at least ten of the countries on the list have documented historical elimination efforts, including Albania [16], Bahrain [17], Chile [18], Greece [16], Japan [19], Jordan [20], Libya [21], Palestine and Israel [22], Tunisia [23], and the former Union of Soviet Socialist Republics (USSR) [24] (Kyrgyzstan, Turkmenistan, and Uzbekistan were certified by the WHO following resurgence in the 1990 s, but their first successful elimination effort under the USSR’s mid-20th century programme was not). The list may thus better be said to enumerate places that no longer have malaria but where the certification process was not undertaken.

Additional countries that have reported achieving zero local malaria cases in at least one year but not yet achieved the criteria to request certification, including Belize, Cabo Verde, Iran, Malaysia, and Timor-Leste, are included in WHO’s E-2020 report [3]. Finally, Feachem et al. independently reviewed 50 historically successful elimination programmes, including six territories not included on the WHO lists [25]: Puerto Rico, Corsica, South Korea, Egypt, Oman, and Syria. Combining territories from across these four lists results in 56 candidate elimination efforts.

### Identifying malaria elimination case series

Annual malaria case series were sought for each of the 56 successful elimination programmes through an extensive non-systematic review of published and unpublished documents, including searches of PubMed (https://pubmed.ncbi.nlm.nih.gov/), Google Scholar (scholar.google.com), Google Books (books.google.com), WHO’s Institutional Repository for Information Sharing (https://apps.who.int/iris), online surveillance databases, and the authors’ existing collection of malaria-related books and reports. Several of these elimination programmes were implemented in regions that today have disputed jurisdictional claims or countries that no longer exist; their inclusion is to understand the impact of the historical programmes implemented there and no statement on their current geopolitical context is intended.

When annual case totals were classified by origin, the number of locally acquired (i.e., autochthonous) cases was recorded separately from the number of imported cases. If not classified (as is typical until case incidence falls to very low levels), all reported cases were assumed to be locally acquired. Where locally acquired cases were further subclassified, indigenous and cryptic cases were tallied together, while introduced or induced cases were not included in case totals since such cases may arise sporadically in response to importation [26] and are not considered to jeopardize elimination [11]. The collated case dataset is available at 10.5281/zenodo0.7250482 [27] as an open source repository that can be updated as additional datapoints are identified.

The first year in which zero indigenous or locally acquired cases were recorded was taken as the termination point of the case series, regardless of whether sporadic or resurgent local transmission occurred subsequently. While this definition of elimination is looser than that required for certification by the WHO (i.e., three years with no indigenous transmission), it permits us to examine a wider range of elimination experiences, including some where success was not maintained. Secondary re-elimination efforts (where a year with zero local cases was achieved, malaria subsequently resurged, and then elimination was again achieved), as in the case of Mauritius [28] or the former republics of the USSR, were not included. Case counts were used directly in analysis rather than converting to rates since in elimination settings case numbers are typically very small compared to overall populations, while highly focalized transmission also makes national figures less useful. Where relevant, case series were truncated to 40 years prior to achievement of zero indigenous cases.

### Analysis

The primary outcome of interest was the number of years required to achieve elimination following initiation of an effective programme intended to achieve that goal. To measure this timeline, the period under which elimination was effectively pursued was differentiated from prior trends that might have decreased case counts due to background factors like socioeconomic improvements, strengthened health systems, urbanization, or land-use changes, among others, or increased them as case management and surveillance systems were intensified. This differentiation was achieved by seeking a breakpoint in the case slope over time, with the assumption that the time from any statistically significant breakpoint to the first year with zero cases demarcated the relevant period during which effective elimination measures were in place.

Breakpoints were identified by first log transforming the annual local case series to enable evaluation of the rate of change in case counts, rather than the absolute changes [29]. A linear slope in cases plotted on the log scale represents a constant rate of decline. For this analysis, the first year with zero cases was replaced with a value of 1 since log 0 is undefined.

Each log transformed series was evaluated for a breakpoint in the linear trend using the *segmented* package in R [30]. The Davies test was used to assess whether any such breakpoint was significant at an alpha of 0.05. If so, the median year of the timeseries was used as a seed value to identify the most likely year at which to segment the time series.

The time to elimination was calculated as the number of years from the breakpoint, if any, to the first year in which zero indigenous cases were reported. In addition, linear regression lines were fit to the two segments separately. The slope from the breakpoint to the first zero year represents the average annual decline rate (r) at which elimination was achieved, which was calculated as r = exp(slope) − 1. Where no significant breakpoint existed, a single linear regression line was fit to all available data and used to calculate the rate of decline, but no timeline was calculated since the first year of the elimination effort could not be identified. The timelines to zero and the associated annual decline rates were summarized across all territories.

### Territory characteristics potentially influencing timelines and annual decline rates

The timeline to elimination and associated annual decline rate were hypothesized to vary depending on characteristics of the territory and its programme. To evaluate these differences, a set of factors were collated for each territory. These included, first, procedural factors including whether the elimination achievement was certified by the WHO, and whether the programme occurred during the Global Malaria Eradication Programme (defined as pre-1980) or the modern era. Second, geographic factors were evaluated including whether the territory is an island or located in the tropics (i.e., centroid located outside 23.436 N or S). Third, sociodemographic factors were assessed, including whether the gross domestic product (GDP) per capita (obtained from [31], using 1950 values for Cyprus, Palestine, and Puerto Rico) in the breakpoint year – or a decade prior to the zero year for territories with no significant breakpoint – or urban fraction of the population (obtained from [32]) was above or below the median. Fourth, factors related to malaria risk were considered including whether baseline case counts were above or below the median and whether the country experienced more than the median importation, measured as the median number of cases reported per year in the five years before and after the zero local case year. The timelines and rates of decline were compared between geographies according to these dichotomized factors of interest using Wilcox-Mann-Whitney tests.

## Results

**Fig. 1:**
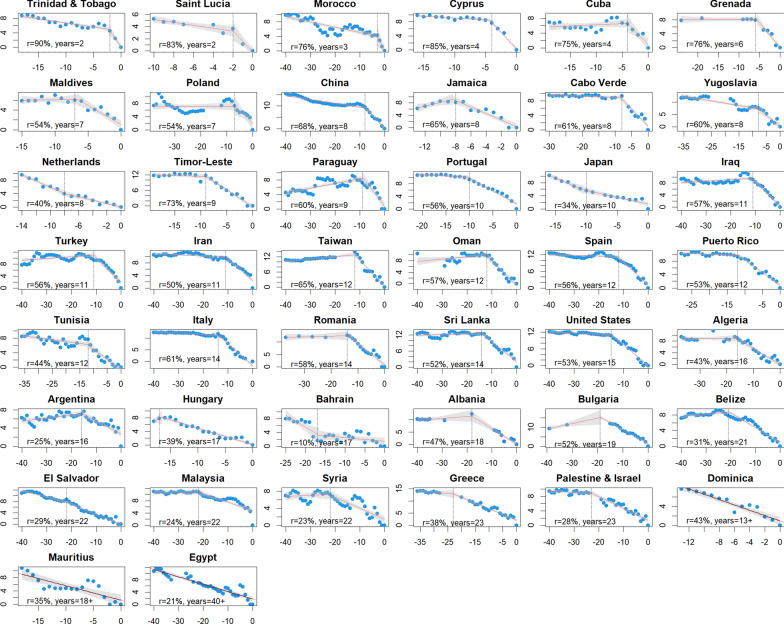
Time series to the first year with zero local or indigenous cases by territory, ordered by elimination timeline length. Linear regression fits are depicted with gray vertical lines marking the significant breakpoint, if any. The x-axis for all panels is years prior to zero cases and the y-axis is the log of the case count. Text in each panel describes the time from the breakpoint to zero and the average annual decline rate over that period

Annual malaria case series leading up to the first zero local case year were compiled for 44/56 of the candidate elimination programmes (Fig. 1). Although many case series were missing occasional years, the overall trends for these 44 territories appeared sufficient to reconstruct the trajectory towards zero. Sufficient classified annual data were not identified to reconstruct the pathway to zero for Australia, Brunei, Chile, Corsica, Jordan, Libya, Reunion, Singapore, South Korea, United Arab Emirates, and Northern Venezuela (the only sub-national region that has received WHO certification).

**Fig. 2:**
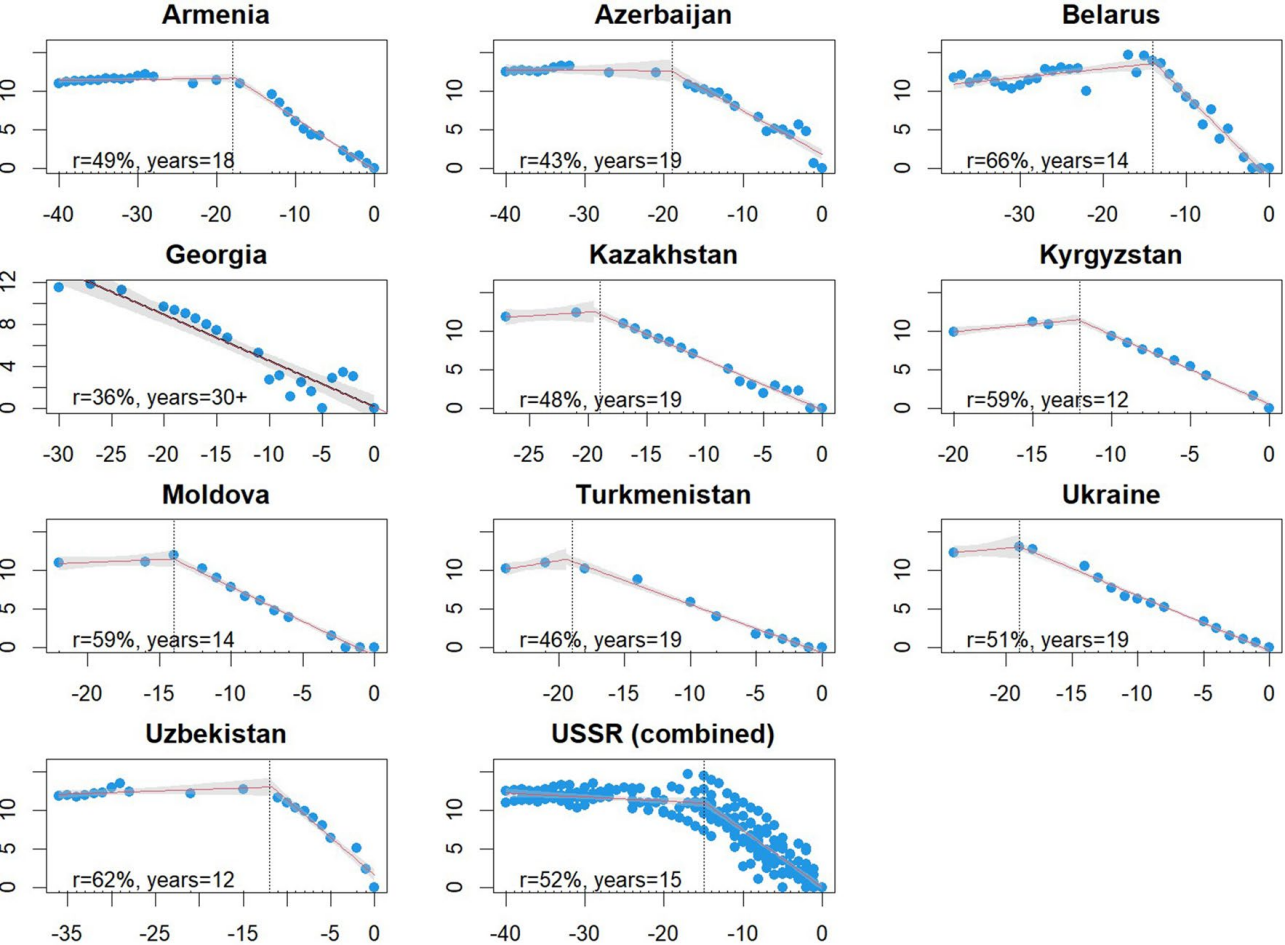
Time series to the first year with zero local or indigenous cases for the former republics of the USSR, individually and combined. Linear regression fits are depicted with gray vertical lines marking the significant breakpoint, if any. The x-axis for all panels is years prior to zero cases and the y-axis is the log of the case count. Text in each panel describes the time from the breakpoint to zero and the average annual decline rate over that period

For the USSR, many decades of incidence data were identified, but no year with zero cases was found during the primary elimination programme of the 1940-1960 s. However, case series ending with a zero-case year were identified for the former republics of Armenia, Azerbaijan, Belarus, Georgia, Kazakhstan, Kyrgyzstan, Moldova, Turkmenistan, Ukraine, and Uzbekistan (Fig. 2). Although Tajikistan reduced local incidence to fewer than ten cases from 1966 to 1968 [33] and later reached zero cases in 2005 [34], a year with zero local cases in the 1960 s was not identified and so it was not included in this analysis. In lieu of a national case series, the breakpoint and associated regression lines were fit to the combined case data from these ten former republics to produce a single average trajectory (the individual case series were not used separately since they shared a common programme conducted across the entire USSR).

Annual importation data could not be found for Tunisia, but it was estimated at 15 cases per year based on an abstract noting that 245 cases were identified over the 16 years following elimination [35]. Specific importation figures were also not identified for Cabo Verde, but median annual importation from 1963 to 1973 was estimated at 30 cases per year from [36].

**Fig. 3:**
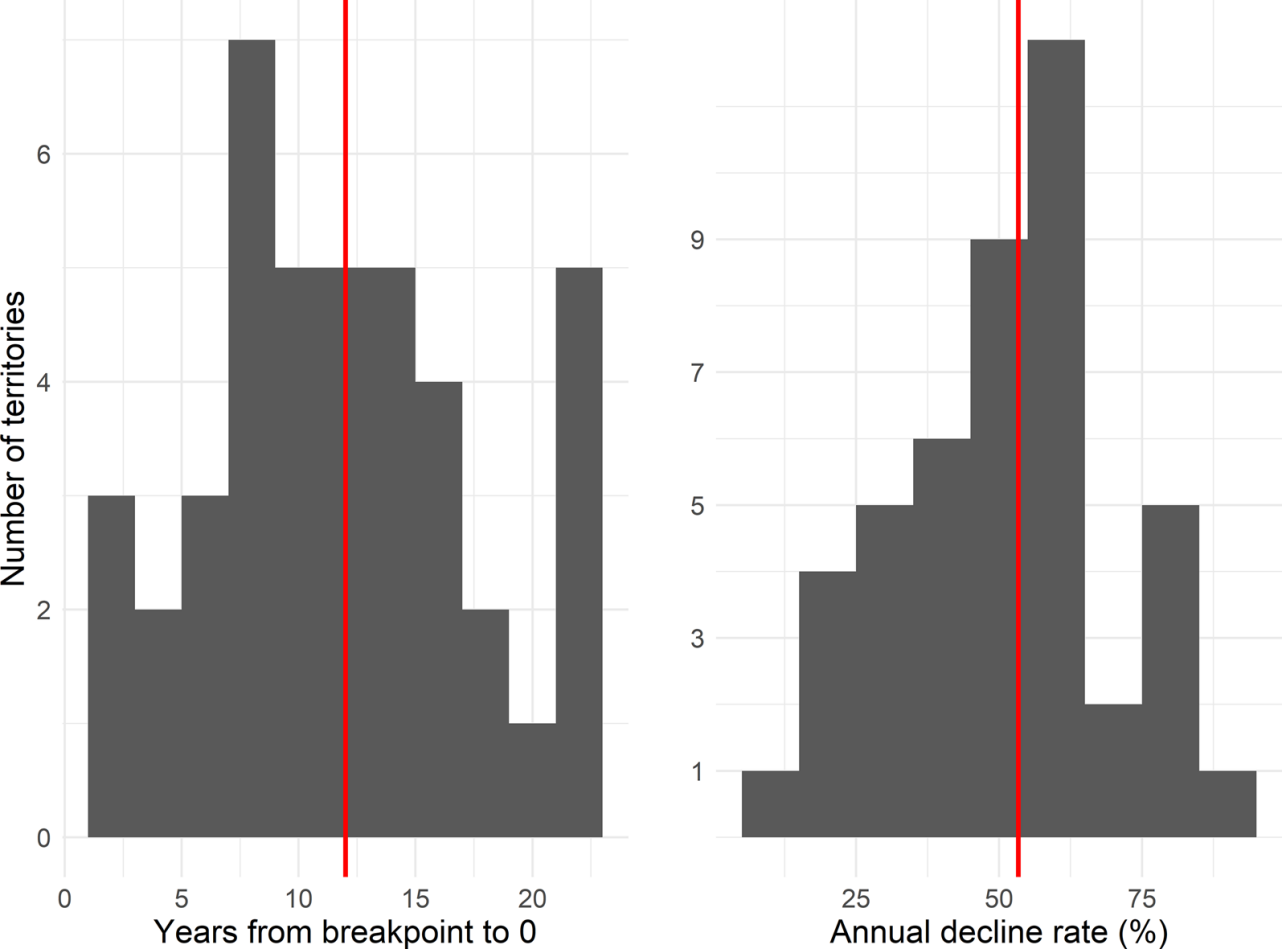
Histograms of (left) time from the fit breakpoint to the first year with zero local cases and (right) the annual decline rate in cases over that period. Red lines represent the medians. Three territories without a significant breakpoint are excluded from the left panel

**Fig. 4:**
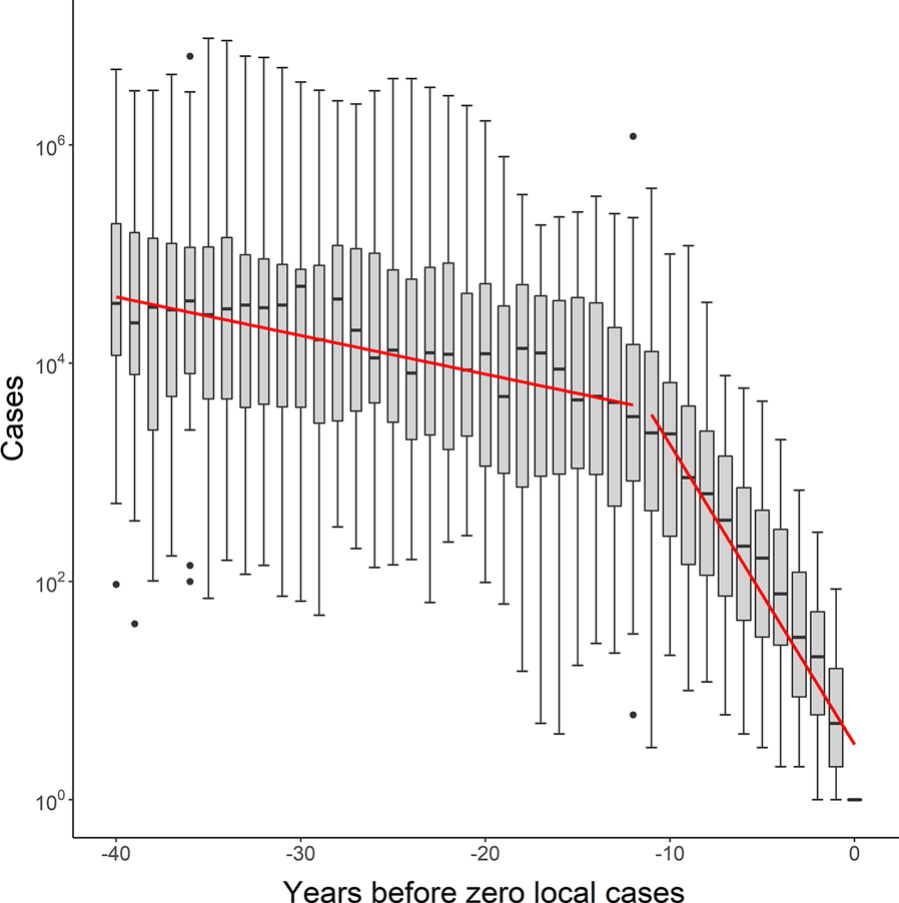
Box-and-whisker plot summarizing the distribution over time of annual local or indigenous case counts across all countries for which time series were identified. The box represents the interquartile range with line showing median, while whiskers indicate maximum and minimum values within 1.5× of the interquartile range and dots outliers beyond this range. Box width is proportionate to the sample size for that year. The red line shows a linear fit segmented at the statistically significant breakpoint calculated for the combined series

Statistically significant breakpoints were identified for 42/45 time series (Figs. 1 and 2), with only Dominica, Egypt, and Mauritius failing to demonstrate one. The median timeline from the breakpoint to the zero year was 12 years (interquartile range of 8–16 years), over which cases declined at a median rate of 53.7% per year (IQR 40.5-60.9%) (Fig. 3). Five years prior to elimination, territories reported a median of 164 cases (IQR 31–449), representing < 1% of their pre-breakpoint average annual total, with a maximum of 4,489 in Cyprus (Fig. 4). Ten years prior to elimination, the median case count was 2,256 (IQR 259-6,657), or around 14% of the pre-breakpoint annual average. Prior to the breakpoint, the median trend was slightly decreasing with median annual decline of r = 2.2% (IQR = decline of 8.9% to increase of 5.3%).

Timelines to elimination differed by a few factors (Table 1). The median time to zero was shorter and the annual decline rate significantly faster among territories where elimination was certified by the WHO (median time to zero = 8.0 years, median r = 56.3%) than those where zero cases were achieved without certification (12 years, r = 45.2%). Island territories (those with no land border with other countries) had significantly shorter (8.0 years) and more rapid (r = 60.8%) declines than non-island territories (14.0 years, r = 52.0%), and territories with centroids in the Tropics tended to achieve elimination more rapidly (8.5 years, r = 55.4%) than non-tropical territories (12.0 years, r = 52.1%), though the difference was not statistically significant. The nine tropical island territories had a median timeline of 7.0 years to elimination compared to 12 years for other territories (W = 249, p = 0.002). Territories that began with more cases took longer to eliminate (12.0 years) than those beginning with fewer (8.0 years). Finally, territories with fewer than the median number of annual imported cases (13 per year) experienced faster declines (r = 57.4%) than those with more importation (50.4%), though timelines were no different.

**Table 1:** Median values of years from breakpoint to zero cases and associated annual decline rate according to a set of binary territory characteristics, with Wilcoxon-Mann-Whitney test statistics

Type of characteristic	Territory characteristic	Years from breakpoint to zero cases			Annual decline rate		
		Yes (n)	No (n)	W, p–value	Yes (n)	No (n)	W, p–value
Procedural	WHO certified	8.0 (25)	12.0 (17)	311, p = 0.012	56.3% (27)	45.2% (18)	359, p = 0.007
	Elimination year before 1980	11.0 (24)	12.0 (18)	260, p = 0.268	54.6% (26)	50.4% (19)	324, p = 0.079
Geographic	No land border (island)	8.0 (13)	14.0 (29)	79.5, p = 0.003	60.8% (15)	52.0% (30)	148, p = 0.065
	Centroid in the tropics	8.5 (14)	12.0 (28)	251.5, p = 0.141	55.4% (16)	52.1% (29)	282, p = 0.244
Socio–demographic	Above median GDP per capita for breakpoint year	12.0 (18)	12.0 (20)	190, p = 0.781	51.1% (20)	55.6% (21)	279, p = 0.074
	% of population in urban areas above median in breakpoint year	12 (21)	10 (21)	283, p = 0.118	50.4% (21)	53.7% (24)	320, p = 0.126
Malaria risk	Average annual pre–breakpoint case count above median	12.0 (21)	8.0 (21)	301.5, p = 0.042	54.5% (22)	43.7% (23)	243, p = 0.831
	Imported cases above the median	11.5 (22)	12.0 (20)	240, p = 0.623	50.4% (23)	57.4% (22)	354, p = 0.021

## Discussion

This review has assembled the most comprehensive set of successful malaria elimination case series to date to evaluate the trajectory with which this goal has been achieved previously. These historical experiences may help countries with aspirations of malaria elimination today to set feasible milestones towards this ultimate goal. Understanding realistic timelines to eliminate is critical for setting plans, since overly optimistic timelines may lead to insufficient funding and long-term planning or community and donor skepticism when targets are missed.

Available data suggest that many countries that have successfully reduced malaria to zero for at least one year have followed similar trajectories. The median pathway to zero involved a 53.7% annual decline in cases over 12 years. This fall to zero was differentiated in almost all case series from a prior phase in which cases tended to trend very slightly downward (with a median annual decline of 2.2%) over many years. This long gradual decline is consistent with the incremental reductions in malaria that might be expected in response to slowly evolving health systems, socioeconomic conditions, urbanization, and environmental change. Tropical island territories such as Trinidad and Tobago, Cabo Verde, and Maldives had the shortest timelines to zero cases.

In 42/45 case series identified, a statistically significant breakpoint was found between these two phases. This review did not seek to identify why each of these breakpoints occurred, or whether specific programmatic changes were required to achieve it, but in many cases it may equate to a programmatic reorientation towards more aggressive elimination-focused efforts [13], such as introduction of indoor residual spray campaigns and expansion of case detection and treatment activities. In the United States, for example, multifactorial changes in socioeconomics, health systems, and environmental modification resulted in decades of sustained declines in malaria incidence [37], from 184,163 cases in 1920 to 61,411 in 1945 [38], an average annual decline of 4.1%. In 1945, a large scale DDT indoor spraying programme was initiated, followed by additional intensive case confirmation and surveillance activities in 1947 [39], which corresponded to the start of a significantly more rapid 53% annual decline in cases from 1946 to 1961. Though beginning these aggressive elimination measures after malaria incidence had already fallen so substantially was belittled as “kicking a dying dog” [40], this substantial acceleration in the case decline rate suggests that the intensified effort played an important role in speeding elimination. If declines had continued at the 1920–1945 rate, over 2,000 cases a year might still be occurring today.

In another example, a breakpoint in the Timor-Leste case series was identified in 2010. Prior to this break, malaria had declined modestly over the prior decade at a rate of 5.9% per year. From 2010, it fell to zero over ten years at a rate of 72.9%. This change in decline rates aligns with a large expansion in the programme, with substantial hiring occurring following increased investment through initiation of the Global Fund’s 2009 round 7 grant, as well as the first implementation of indoor residual spraying in three districts as a supplement to bed nets [41]. Similarly, in Cyprus, the breakpoint found in 1947 follows the organization of the “*Anopheles* (Malaria) Eradication Campaign” initiated in 1946 [42], and in Oman, the 1992 breakpoint follows the 1991 pilot launch of a National Malaria Eradication Programme which conducted larviciding and early case detection and treatment [43].

The case studies examined here represent only those territories that successfully achieved the endpoint of zero annual local cases. Many other countries have sought but to date failed to achieve this aim: they thus represent longer, uncompleted trajectories. Because this review considers only successful elimination case series, the results manifest a survivorship bias. They should, therefore, not be interpreted as average results for all countries, but they illustrate best case scenarios of what has been achievable to date. Conversely, future elimination trajectories should not be limited by malaria’s history. Improvements in surveillance and technology, budget increases, and more effective tools could all enable elimination progress to occur more rapidly. More targeted approaches that effectively engage communities in focal high transmission areas (typically remote regions with poor access to quality healthcare) could also accelerate timelines. Setting more ambitious timelines than what has been observed here thus is not necessarily inappropriate. Nevertheless, historical precedent offers a sense of what has been possible to date.

Anticipating that elimination may require maintaining efforts over at least a decade of low incidence will mean adopting methods for sustaining elimination programmes even as malaria is no longer a large burden on public health [44]. Doing so efficiently and sustainably will likely require the malaria community to work towards incorporation of malaria services into stronger health systems, rather than working through independent channels that will be hard to maintain as malaria becomes less of a visible threat. For example, integration of vertical, malaria-focused programmes like village malaria workers into broader primary healthcare strategies [45] and malaria-specific surveillance systems into comprehensive disease information systems may help improve sustainability and thus the probability of success. Given the potential for long timelines to elimination, malaria programmes may also need to consider intermediate milestones that they can achieve and celebrate en route to the ultimate goal of elimination; Cambodia, for example, has successfully reduced malaria mortality to zero since 2018, aims to eliminate *Plasmodium falciparum* by 2023, and has an ultimate goal of eliminating all human malaria by 2025 [46].

The historical case data used here are necessarily incomplete and subject to numerous problems. Over the course of an elimination programme, observed case numbers will change due to reasons other than true changes in transmission. The typical arc of an elimination programme may involve phases with decreased malaria-attributable incidence as acceptance of clinical diagnosis or suspected malaria shifts to a requirement for confirmation of all cases, increases as quality case management services are extended to those who need it, and finally an exponential fall in cases as effective prevention tools are targeted to high transmission areas [11]. Case counts may also manifest background trends over time due to factors that influence transmission, including socioeconomic improvements, urbanization, changes to the built environment, and environmental changes [47]. Case numbers may also fall if cases begin to be classified by origin in a place where a substantial fraction are actually due to importation or introduction. The trajectories collated here are thus not necessarily representative of true changes in malaria so much as how malaria is tallied over time, and these case series must be interpreted as the product of all of those evolutions. Nevertheless, the median declines from the breakpoints to zero cases align reasonably well with modelled elimination timelines [10].

This analysis was limited to fitting a single breakpoint to each case series. Visual inspection of the resulting linear fits in Fig. 1 suggests this approach adequately describes many, but not all of the case series collated here. In many territories, case series manifest a variety of increases and decreases over time which could be captured by fitting more breakpoints or through more complex modelling approaches. The parsimonious approach of limiting analysis to a single breakpoint enables us to summarize generalized pathways to elimination across all countries, but this simplification is inadequate to fully understand the variety of reasons why countries may have seen incidence change over time.

High rates of importation may continually replenish the infectious reservoir and maintain endemic transmission [48, 49]. Territories that successfully eliminated malaria were found here to have reported only a median of 13 imported cases (IQR 2–31) annually at the time elimination was achieved. Only six territories (Turkey, Bahrain, Malaysia, Oman, Iran, and China) were reported to have more than a median of 100 imported cases per year, and only China had over 1,000 (n = 3,022). These results suggest importation could be a particular challenge for countries in sub-Saharan Africa seeking to eliminate, since imported cases rates there are typically far higher than observed in these successful historical examples: Namibia, for example, reported almost 12,000 imported cases in 2017, and South Africa reported almost 9,000 in 2019 [34]. Devising elimination plans over interconnected zones linked by mobility rather than national boundaries may be essential for elimination to be feasible in such a setting [50].

## Conclusion

Results of this review suggest that malaria elimination has been achieved over widely varying trajectories, with a median timeline of 12 years. Setting goals for malaria elimination on shorter timescales may be most appropriate in isolated, low importation settings such as islands, while other regions aiming to eliminate malaria need to consider how best to maintain vital case management and vector control services over substantially longer timescales. Given the potential for elimination to require many years of sustained effort, elimination planning should strategically integrate anti-malaria efforts within the larger health ecosystem to ensure ongoing suppression of transmission until zero cases are reached. An evidence-based understanding of timelines to elimination in specific contexts can allow creation of realistic financial and operational plans, which will be vital to ensure sufficient funding, political will, and community support to secure additional momentous successes against malaria in the future.

## Data Availability

The datasets generated during the current study are available in the Zenodo repository, 10.5281/zenodo.7250482. Additional publicly available datasets analysed in this investigation are cited in the text.

## Abbreviations

GDP: Gross domestic product
IQR: Interquartile range
USSR: Union of Soviet Socialist Republics
WHO: World Health Organization

## References

[1] Feachem RGA, Chen I, Akbari O, Bertozzi-Villa A, Bhatt S, Binka F (2019). Malaria eradication within a generation: ambitious, achievable, and necessary. Lancet.

[2] Lindblade KA, Li Xiao H, Tiffany A, Galappaththy G, Alonso P, Abeyasinghe R (2021). Supporting countries to achieve their malaria elimination goals: the WHO E-2020 initiative. Malar J.

[3] WHO (2021). Zeroing in on malaria elimination: final report of the E-2020 initiative.

[4] Andrada A, Herrera S, Yé Y (2019). Are new national malaria strategic plans informed by the previous ones? A comprehensive assessment of sub-Saharan African countries from 2001 to present. Malar J.

[5] Whitty CJM (2015). Political, social and technical risks in the last stages of disease eradication campaigns. Int Health.

[6] Wickett JF (2002). The Final Inch: The Eradication of Smallpox and Beyond. Soc Sci.

[7] Black RH (1968). Manual of epidemiology and epidemiological services in malaria programmes.

[8] Pampana E (1969). Textbook of malaria eradication.

[9] Macdonald G, Goeckel GW (1964). The malaria parasite rate and interruption of transmission. Bull World Health Organ.

[10] Smith DL, Hay SI (2009). Endemicity response timelines for *Plasmodium falciparum* elimination. Malar J.

[11] WHO (2017). A framework for malaria elimination.

[12] Feachem RG, Phillips AA, Targett GA (2009). Shrinking the malaria map: a prospectus on malaria elimination.

[13] Moonen B, Cohen JM, Snow RW, Slutsker L, Drakeley C, Smith DL (2010). Operational strategies to achieve and maintain malaria elimination. Lancet.

[14] WHO. Preparing for certification of malaria elimination. Geneva: World Health Organization; 2020. https://doi.org/https://www.who.int/publications-detail-redirect/9789240005624. Accessed 21 Mar 2022.

[15] WHO. Countries and territories certified malaria-free by WHO. Geneva: World Health Organization; 2022. https://doi.org/https://www.who.int/teams/global-malaria-programme/elimination/countries-and-territories-certified-malaria-free-by-who. Accessed 21 Mar 2022.

[16] Bruce-Chwatt LJ, de Zulueta J (1980). The rise and fall of malaria in Europe: a historico-epidemiological study. English language ed.

[17] Mahmood R (1992). History of eradication of malaria in Bahrain. J Bahrain Med Soc.

[18] Schenone H, Olea A, Rojas A, García N (2002). [Malaria in Chile: 1913–2001](in Spanish). Rev Med Chil.

[19] WHO. Information on the malaria control programme in Japan. World Health Organization; Report No.: WHO/Mal/103.23. 1953. https://doi.org/http://apps.who.int/iris/bitstream/handle/10665/64277/WHO_Mal_103.23.pdf. Accessed 17 July 2021.

[20] WHO. Information on the malaria control programme in Jordan. World Health Organization. Report No.: WHO/Mal/163-13. 1956. https://doi.org/https://apps.who.int/iris/bitstream/handle/10665/64484/WHO_Mal_163-13.pdf. Accessed 9 May 2022.

[21] Goodwin W. Oasis malaria in Libya. Report No.: EM/ME-Tech.2/8. 1959. https://doi.org/https://applications.emro.who.int/docs/EM_ME_Tech_2_8_EN.pdf. Accessed 18 July 2021.

[22] Kligler IJ (1930). The epidemiology and control of malaria in Palestine.

[23] Ambroise-Thomas P, Wernsdorfer W, Grab B, Cullen J, Bertagna P. Longitudinal sero-epidemiological studies on malaria in Tunisia. World Health Organization; Report No.: WHO/MAL/74.834. 1974. http://apps.who.int/iris/bitstream/handle/10665/65701/WHO_MAL_74.834.pdf. Accessed 18 July 2021.

[24] Bruce-Chwatt LJ (1959). Malaria research and eradication in the USSR. Bull World Health Organ.

[25] Feachem R, Phillips AA, Hwang J, Cotter C, Wielgosz B, Greenwood BM (2010). Shrinking the malaria map: progress and prospects. Lancet.

[26] Cohen JM, Moonen B, Snow RW, Smith DL (2010). How absolute is zero? An evaluation of historical and current definitions of malaria elimination. Malar J.

[27] Cohen JM. How long is the last mile? Evaluating successful malaria elimination trajectories [Dataset] (1.2) [Internet]. Zenodo; 2022. https://doi.org/https://zenodo.org/record/7250482. Accessed 1 Nov 2022. 10.1186/s12936-022-04368-3 PMC9664685 36376935

[28] Tatarsky A, Aboobakar S, Cohen JM, Gopee N, Bheecarry A, Moonasar D (2011). Preventing the reintroduction of malaria in mauritius: a programmatic and financial assessment. PLoS ONE.

[29] Swaroop S, Gilroy AB, Uemura K, Organization WH, others. Statistical methods in malaria eradication. 1966. https://doi.org/http://apps.who.int/iris/handle/10665/41775. Accessed 5 July 2022. 4955782

[30] Muggeo VMR. segmented [Internet]. 2022. https://doi.org/https://cran.r-project.org/web//packages/segmented/segmented.pdf. Accessed 6 Apr 2022.

[31] Bolt J, van Zanden JL. Maddison style estimates of the evolution of the world economy. A new 2020 update [Internet]. 2020. https://doi.org/https://www.rug.nl/ggdc/historicaldevelopment/maddison/releases/maddison-project-database-2020. Accessed 21 Apr 2022.

[32] UN, Department of Economic and Social Affairs, Population Division. World Urbanization Prospects 2018, Online Edition [Internet]. Geneva: United Nations Population Division; 2018. https://doi.org/https://population.un.org/wpp/Download/Standard/Population/. Accessed 10 May 2022.

[33] Sergiev PG, Dukhanina NN, Sarikian SI, Zhukova TA (1969). [Malaria in the USSR from 1966 to 1968 and immediate tasks for its prevention](in Russian). Med Parazitol (Mosk).

[34] WHO. World malaria report 2021. Geneva: World Health Organization; 2021. https://doi.org/https://apps.who.int/iris/handle/10665/350147. Accessed 14 Jan 2022.

[35] Bouratbine A, Chahed MK, Aoun K, Krida G, Ayari S, Ben Ismail R [Imported malaria in Tunisia](in French). Bull Soc Pathol Exot 1990. 1998;91:203–7. 9773190

[36] Ministry of Health Cape Verde. Organization WH, University of California, San Francisco. Eliminating Malaria Case-study 2: Moving towards sustainable elimination in Cape Verde. Geneva: World Health Organization; 2012. https://doi.org/https://apps.who.int/iris/handle/10665/204372. Accessed 21 Apr 2022.

[37] Andrews JM (1948). What’s happening to malaria in the USA?. Am J Public Health.

[38] Medical, Division (1945). Malaria Control in War Areas, US Public Health Service. Epidemiological data for malaria control activities.

[39] Andrews JM (1951). Nation-wide malaria eradication projects in the Americas. I. The eradication program in the U.S.A. J Natl Malar Soc.

[40] Humphreys M (1996). Kicking a dying dog: DDT and the demise of malaria in the American South, 1942–1950. Isis.

[41] Yapabandara MA, Sarmento R, de Fatima Mota M, do don Bosco R, Martins J, Wickremasinghe NAR. Evidence-based malaria control in Timor Leste from, (2006). Evidence-based malaria control in Timor Leste from 2006 to 2012. Malar J..

[42] Constantinou K (1998). *Anopheles* (malaria) eradication in Cyprus. Parassitologia.

[43] Department of Health Information & Statistics; Directorate of General Planning; Sultanate of Oman. Annual Health Report 2020. Muscat: Sultanate of Oman; 2020.

[44] CHAI, UCSF, ALMA. Maintaining the gains in global malaria control. Boston: Clinton Health Access Initiative, University of California San Francisco, and the African Leaders Malaria Alliance; 2011.

[45] Napier HG, Baird M, Wong E, Walwyn-Jones E, Garcia ME, Cartagena L (2021). Evaluating vertical malaria community health worker programs as malaria declines: learning from program evaluations in Honduras and Lao PDR. Glob Health Sci Pract.

[46] WHO (2022). The Mekong Malaria Elimination Programme. Accelerating malaria elimination in the Greater Mekong, Bulletin 10, March 2022.

[47] WHO (2014). From malaria control to malaria elimination: a manual for elimination scenario planning.

[48] Guerra CA, Kang SY, Citron DT, Hergott DEB, Perry M, Smith J (2019). Human mobility patterns and malaria importation on Bioko Island. Nat Commun.

[49] Le Menach A, Tatem AJ, Cohen JM, Hay SI, Randell H, Patil AP (2011). Travel risk, malaria importation and malaria transmission in Zanzibar. Sci Rep.

[50] Tatem AJ, Smith DL (2010). International population movements and regional *Plasmodium falciparum* malaria elimination strategies. Proc Natl Acad Sci USA.

